# Hydatid cyst ruptured in the biliary duct: an exceptional cause of acute pancreatitis

**DOI:** 10.11604/pamj.2014.18.298.5147

**Published:** 2014-08-14

**Authors:** Ahmed Belkouch, Abdelilah Mouhsine

**Affiliations:** 1 Emergency Department, Military Hospital of Instruction Mohamed V, Rabat, Morocco; 2 Radiology service, Military hospital Avicenna, Marrakech, Morocco

**Keywords:** Hydatid cyst, biliary duct, acute pancreatitis

## Image in medicine

A 49 years old man presented to the emergency department complaining of abdominal pain. He had no medical history and no alcoholism or drug consumption. The pain started 2 days before, accompanied by vomiting and without bowel disturbances. On examination the patient was jaundiced, conscious, cooperative, afebrile, the heart rate was at 110 beats / min, Blood Pressure at 160/82 mmHg, respiratory rate was 25c/min. The pain was epigastric without special irradiation, laboratory data demonstrated a high level of serum lipase: 1199U/L (normal 22-51U/L), total bilirubin 68 mg/l (normal 3-12mg/L), alanine aminotransferase, 183U/L (normal 14-54U/L); aspartate aminotransferase, 105 U/L (normal 15-41 U/L); alkaline phosphatase, 306U/L (normal 32-91U/L); gamma glutamyl trans peptidase 656 U/L (normal 7-50U/L), creatinine, urea levels and Complete blood count were normal. The MRI showed an edematous pancreas (A), a large Hydatid cyst of the left hepatic lobe in contact with the left bile duct (B, C). The common bile duct was dilated (15mm) (D). After pain treatment and correction of hydro electrolytic disturbances, we performed endoscopic retrograde cholangio pancreatography which showed dilated wrung and the absence of opacification of the left hepatic biliary duct (E). Immediately after endoscopic sphincterotomy, hydrated membranes and small vesicles were seen protruding spontaneously through the papillary orifice (F). The patient was discharged from the hospital 10 days later after clinical and biological resolution.

**Figure 1:**
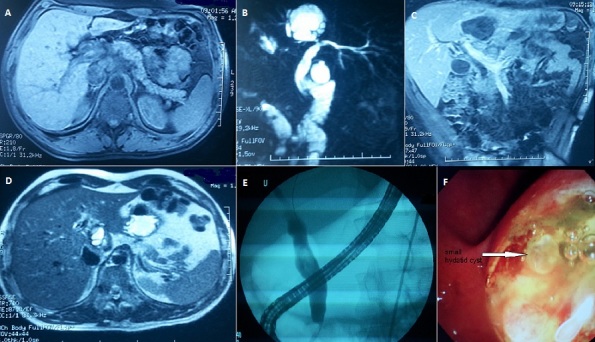
A) axial T1 weighted acquisition showing the épaississment and cephalic infiltration of the pancreas including the biliopancreatic duct with upstream dilatation of the common bile duct; B) bili-MRI sequence showing the contact between the hydatid cyst and the left branch of the bile duct; C)coronal T1-weighted acquisition sequence showing a hydatid cyst of the left lobe of the liver in contact with the dilated left branch of the bile duct; D) axial T2-weighted acquisition sequence showing the dilated CBD; E)Endoscopic retrograde cholangio pancreatography showing dilated common bile duct with defect image and absence of opacification of the left hepatic duct; F) Endoscopic sphincterotomy and issue of hydatid vesicles

